# Integrated computational screening and liquid biopsy approach to uncover the role of biomarkers for oral cancer lymph node metastasis

**DOI:** 10.1038/s41598-023-41348-2

**Published:** 2023-08-28

**Authors:** Shayma Shaikh, Deep Kumari Yadav, Kinjal Bhadresha, Rakesh M. Rawal

**Affiliations:** 1 Department of Life Science, School of Sciences, Gujarat University, Ahmedabad, Gujarat 380009 India; 2 Department of Biochemistry and Forensic Science, School of Sciences, Gujarat University, Ahmedabad, Gujarat 380009 India; 3 National Institute of Health, Bethesda, MD USA

**Keywords:** Oral cancer detection, Tumour angiogenesis

## Abstract

Cancer is an abnormal, heterogeneous growth of cells with the ability to invade surrounding tissue and even distant organs. Worldwide, GLOBOCAN had an estimated 18.1 million new cases and 9.6 million death rates of cancer in 2018. Among all cancers, Oral cancer (OC) is the sixth most common cancer worldwide, and the third most common in India, the most frequent type, oral squamous cell carcinoma (OSCC), tends to spread to lymph nodes in advanced stages. Throughout the past few decades, the molecular landscape of OSCC biology has remained unknown despite breakthroughs in our understanding of the genome-scale gene expression pattern of oral cancer particularly in lymph node metastasis. Moreover, due to tissue variability in single-cohort studies, investigations on OSCC gene-expression profiles are scarce or inconsistent. The work provides a comprehensive analysis of changed expression and lays a major focus on employing a liquid biopsy base method to find new therapeutic targets and early prediction biomarkers for lymph node metastasis. Therefore, the current study combined the profile information from GSE9844, GSE30784, GSE3524, and GSE2280 cohorts to screen for differentially expressed genes, and then using gene enrichment analysis and protein–protein interaction network design, identified the possible candidate genes and pathways in lymph node metastatic patients. Additionally, the mRNA expression of discovered genes was assessed using real-time PCR, and the Human Protein Atlas database was utilized to determine the protein levels of hub genes in tumor and normal tissues. Angiogenesis was been investigated using the Chorioallentoic membrane (CAM) angiogenesis test. In a cohort of OSCC patients, fibronectin (FN1), C-X-C Motif Chemokine Ligand 8 (CXCL8), and matrix metallopeptidase 9 (MMP9) were significantly upregulated, corroborating these findings. Our identified significant gene signature showed greater serum exosome effectiveness in early detection and clinically linked with intracellular communication in the establishment of the premetastatic niche. Also, the results of the CAM test reveal that primary OC derived exosomes may have a function in angiogenesis. As a result, our study finds three potential genes that may be used as a possible biomarker for lymph node metastasis early detection and sheds light on the underlying processes of exosomes that cause a premetastatic condition.

## Introduction

One of the major problems for community health is oral cancer (OC), which is the sixth most prevalent cancer overall and the third most prevalent in India with around 77,000 incidences and 52,000 fatalities recorded there. Almost 90% of OCs are squamous cell carcinomas (SCC), which develop from the epithelium lining of the oral cavity. At the time of diagnosis, more than 50% of OSCC patients have metastases to nearby lymph nodes^1^. Regional lymph node metastasis is a significant prognostic indicator for choosing the best course of treatment. Patients with lymph node metastasis at presentation had a 5-year survival rate of 25–40%, compared to about 90% for patients who do not have metastasis^2^. Regrettably, such localized or distant metastases of OSCC are typically thought to be resistant to standard medical care^3^ Therefore, the early detection of lymph node metastases is clinically important for better clinical outcomes in OC patients.

Extracellular vesicles (EVs), such as exosomes, 30 to 150 nm vesicles^4^ that mediate cell-to-cell communication, are one possible strategy for more sensitive detection of cancer-related biomarkers from blood^5^. Exosomes have been demonstrated to be released by certain cancers into the bloodstream, where they carry functional protein, mRNA and miRNA biomarkers that describe the tumor^6^. Exosomes from cancer cells have been shown to affect angiogenesis, immunology, and metastasis through biological function investigation, which has a significant impact on promoting carcinogenesis^7^. Recently, it has been proposed that by employing various methods, such as microarray for various cancers, it may be possible to more accurately diagnose cancer using EV-bound mRNA biomarkers^8^. Three metastasis-specific markers, secreted phosphoprotein 1(SPP1), cell surface adhesion receptor (CD44), and Periostin (POSTN), were recently effectively found in serum-derived EVs by Kinjal et al., demonstrating the potential role of exosomes for early prediction^9^. Exosomal protein markers such as TNF Receptor Associated Protein 1(TRAP1), epidermal growth factor receptor (EGFR), heat shock protein 90 (HSP-90), and Matrix Metallopeptidase 13 (MMP-13), which can modify gene intracellular activities, have been identified as having potential for early diagnosis of OSCC^10^. Therefore, it is crucial to identify accurate gene signatures or molecular biomarkers for lymph node metastases from oral cancer in order to use them as diagnostic or prognostic tools in routine OC clinical management.

As a result, the aim of this work is to employ bioinformatics methods to discover DEGs and pathways that contribute to OC metastasising to lymph nodes. In order to fulfil the meta-analysis perspective of the study, multiple microarray datasets were used. Further, to understand the critical role of exosomes—a liquid biopsy component- in OC metastasis, we also analysed the gene expression of exosomes obtained from primary and metastatic OC. The CAM metastasis experiment was also carried out on chick embryos. It is a very convenient and cost-effective animal model to study the mechanism of tumor metastasis^11^.

## Materials and methods

### Data analysis using GeneSpring

Gene expression Omnibus (GEO) (NCBI, http://www.ncbi.nlm.nih.gov/geo/), a public functional genomic data repository was used to obtain the pre-existing raw microarray datasets. These datasets were searched using the following criteria: “OSCC”, “OC”, “Oral Cavity”, “Lymph node metastasis”, “metastasis”, “Expression profiling by array”, and Sample count > 10. Datasets having cell lines and HPV positive were excluded from the study. Four datasets GSE9844 (https://www.ncbi.nlm.nih.gov/geo/query/acc.cgi?acc=GSE31056)^12^, GSE30784 (https://www.ncbi.nlm.nih.gov/geo/query/acc.cgi?acc=GSE30784)^13^, GSE3524 (https://www.ncbi.nlm.nih.gov/geo/query/acc.cgi?acc=GSE3524)^14^ and GSE2280 (https://www.ncbi.nlm.nih.gov/geo/query/acc.cgi?acc=GSE2280)^15^ were included in the present analysis to find out key genes regulating oral cancer metastasise to Lymph nodes (Table 1). The raw data files were downloaded as. CEL format from the GEO database and analysed by using GeneSpring software (http://genespring-support.com). Briefly, the raw data was uploaded to GeneSpring software, where the samples were baseline transformed and normalised using Affymetrix's Robust Multiarray Analysis (RMA). The isolated sample files were then divided into three categories: ‘Control’ 'Primary' and 'Metastases,' and examined as a single experiment. Principal Component Analysis (PCA) in GeneSpring was used to create experimental data at the gene level (arithmetic mean of all probes corresponding to the same probe ID)^16^. To identify DEGs between groups, a Student's t-test was used. log fold-change > 2 and adjusted Benjamini–Hochberg corrected *P* < 0.05 were used as cut-off criteria. The integrated pathway analysis tool was also used to analyse the discovered gene pathways. The GeneSpring programme was used to extract the individual gene entity lists and export them to Excel files (Supplementary File 1).

**Table 1:** Datasets retrieved from gene expression omnibus for LN metastasis.

No	Datasets	Array platform	Site detail	Pubmed ID	No. of cases	No. of controls
1	GSE9844	Affymetrix Human Genome U133 Plus 2.0 Array	Oral tongue squamous cell carcinoma (OTSCC)	200009844	26	12
2	GSE30784	Affymetrix Human Genome U133 Plus 2.0 Array	Oral cavity SCC	200030784	167	45
3	GSE3524	Affymetrix Human Genome U133A Array	Oral cavity SCC to LN metastasis	1584	16	04
4	GSE2280	Affymetrix Human Genome U133A Array	Squamous cell carcinoma of the oral cavity (OSCC) to LN metastasis	200002280	22	05

### Identification of hub genes responsible for OC to LN metastasis

To identify the gene signature pattern for OC to LN metastasis, up- and down-regulated genes between primary and metastasis were compared using Venn diagrams. Moreover, the Functional Enrichment Analysis program STRING was employed to identify functional classes in protein–protein interaction networks. Additionally, the CytoHubba plugin of Cytoscape Software identified the top 10 hub genes based on bottleneck, degree, and closeness, and the top three genes shared by these three characteristics were regarded as hub genes and adopted for further validation.

### Gene ontology analysis and signaling pathway enrichment of DEGs

Gene Ontology (GO) analysis is an important method for annotating genes and determining biological properties such as cellular component (CC), molecular function (MF), and biological process (BP). The Kyoto Encyclopaedia of Genes and Genomes (KEGG) pathway enrichment analysis was used to investigate the role of DEGs in various pathways^17^.

Shiny GO (http://bioinformatics.sdstate.edu/go/) A Gene Ontology tool was used for CC, MF, BP and KEGG pathway Analysis of hub genes.

### Validation of key potential DEGs using patient’s sample

**Figure 1:**
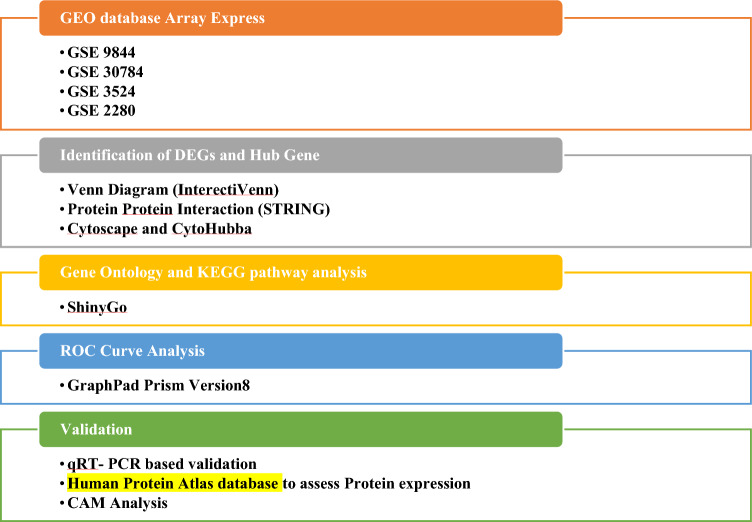
Workflow for identifying a panel of genes associated with lymph node metastasis as biomarkers.

Tumor tissues (n = 20) and serum samples from OC patients (n = 30) with LN metastasis (n = 10) and without LN metastasis (n = 20) were obtained from Apollo Hospital, Ahmedabad with their informed written consent. 10 Healthy Controls were enrolled for this study. The validation of genes predicted from the bioinformatics meta-analysis was done by serum derived exosomal and tumor tissue derived RNA using quantitative real-time PCR. This study is ethically approved by the human ethical committee at Gujarat University under certification number/reference number GU/IEC/10/2018 (Fig. 1).

### Isolation of serum derived exosomes from primary and metastatic patients

Exosomes were isolated using Total exosomal isolation Reagent (from Serum, Invitrogen) from serum of primary and LN metastatic patients. Briefly, 200 µl of serum sample were mixed with 40 µl of the Total Exosome Isolation reagent. Mixture was vortexed, homogenised and incubated at 2 °C to 8 °C for 30 min. After incubation, samples were centrifuged at 10,000 × g for 10 min at room temperature. The supernatant was discarded and the cell pellet was resuspended in a convenient volume in 1 × PBS and stored at − 20 °C until further analysis.

### Characterisation of exosomes

#### Nanoparticle tracking analysis (NTA)

The size distribution profile of exosomes was calculated using a NanoSight NS300 (Malvern Instruments, Malvern, UK) equipped with 532 nm laser. (ZEN0118, Malvern Instruments, Malvern, UK). This method is based on Brownian motion of nanoparticles to track their individual moments and extract relevant data^18^. NTA software (version 2.3; NanoSight Ltd., Amesbury, UK) was used to evaluate particle movement. Samples were diluted 1:1000 in PBS to achieve a quality particle size distribution. Three 30-s videos were recorded at a frame rate of 30 frames per second for each sample.

#### FE-SEM

Exosome imaging requires the use of very high-resolution microscopy due to their small size. Field Emission Scanning Electron Microscope (FE-SEM) is an imaging tool that enables the analysis of conductive, non-conductive, and high-vacuum incompatible materials by scanning the sample surface with a high-energy electron beam in a raster scan pattern. In both standard high vacuum and environmental modes, it provides nanoscale resolution and a good signal-to-noise ratio. The EDS has a new 80 mm^2^ SDD detector that allows for high-resolution element detection. FEI Quanta 200 F SEM (FEI Company (Netherland)) was used to analyse the size of exosome.

#### Flow cytometry

Data was collected using a BD FACSCalibur flow cytometer without any cell sorting. Flow cytometry is a molecular approach used to characterize exosomal surface proteins. Exosome-specific markers CD63, CD81 and CD9 were analysed for characterisation. Data were obtained using 2 individual apogee A50/Micro flow FCs equipped with 50 mW 405-nm (violet), 488-nm (blue), and 638-nm (red) lasers^19^. The samples were diluted (final dilution 1:25) with 480 µl of 0.22 µm pre-filtered PBS and were incubated in 10 µl of mouse anti-CD63- Alexa 488, CD-9 APC, and CD-81PE antibodies for 60 min.

### RNA extraction and qRT-PCR

Total RNA was isolated with TRIzol reagent (Invitrogen; Thermo Fisher Scientific, Inc.) according to the manufacturer’s procedure. Further, RIPA buffer was used to lyse the exosome prior to the RNA extraction. Nanodrop was used to determine the purity of the RNA using the 260/280 ratio (Epoch BioTek system). A total of 1 µg of RNA was converted into complementary DNA (cDNA) using Bio-Rad cDNA Synthesis kit following the manufacturer's protocol.

For Real-Time PCR briefly, 20 µl volume that included 10 µl SYBR Green QPCR Master Mix (QuantiNova SYBR Green, Cat No./ID: 208052) containing 0.5 µl (200 nM) each of specific forward & reverse primer and 2 µl cDNA as template. GAPDH1 was used as a housekeeping gene in each experiment set. The detailed sequence of primers is shown in Table 2.

**Table 2:** Detailed list of primers.

Sr. no	Gene name	Sequence	No. of base
1	FN1	FP 5’-CTGTGACAACTGCCGCAGAC-3’	20
		RP 5’-CGGGAATCTTCTCTGTCAGCCT-3’	22
2	CXCL8	FP 5’-ACACTGCGCCAACACAGAAA-3’	20
		RP 5’-TTCTCAGCCCTCTTCAAAAACTTC-3’	24
3	MMP9	FP 5’-CATCCAGTTTGGTGTCGCGG-3’	20
		RP 5’-CGTCATCGTCGAAATGGGCG-3’	20
4	GAPDH	FP 5’-ACTTTGGTATCGTGGAAGGACTCAT-3’	25
		RP 5’-GTTTTTCTAGACGGCAGGTCAGG-3’	23

Quantitative PCR using Sybr green chemistry was performed in a 96-well reaction plate format in QuantStudio5 (Applied Biosystems, USA, CA) under the following thermal cycling conditions: 1 cycle of 10 min at 95 degrees Celsius for initial denaturation, 40 cycles of 15 s at 95 degrees Celsius for denaturation, 1 min at 60 degrees Celsius for annealing and extension, followed by melting curve detection to ensure positive amplification of the target gene rather than non-specific products or primer dimmers. The ΔΔCT or 2^ΔΔCT^ method was used to determine the fold change expression. The average CT value was derived for the quantification of fold change analysis after each experiment was done in triplicate independently^20^.

### ROC curve analysis

The receiver operating characteristic (ROC) curve was created using GraphPad Prism version 8 (GraphPad Software, USA). ROC curves were developed to evaluate the predictive potential of each biomarker validated by qRT-PCR analysis. By numerical integration of sensitivity and specificity the ROC curve creates the area under the curve (AUC), which demonstrated the markers’ abilities. Higher AUC values, which range from 0.5 to 1.0, indicate better biomarkers. As a result, an AUC score closes to 1.0 denotes perfect biomarker competence^21^.

### Protein level of hub genes in HPA database

Furthermore, the HPA database (https://www.proteinatlas.org/), which contains immunohistochemistry-based expression data for specific human tissues, was used to assess the protein levels of hub genes in tumor and normal tissues^22^.

### Chorioallentoic membrane (CAM) angiogenesis assay

The CAM experiments proceeded according to a previously described protocol with minor modifications^17^. Briefly, fertilized 6th-day Rhode Island red hen eggs were received from animal husbandry (Intensive poultry development block, Makarba, Ahmedabad, Gujarat, India) and incubated for 48 h at 37 °C with 60% humidity.

On day 8, the small window was created carefully allowing the yolk sack blood vessels to be faced upwards. Silicone O-rings with an inside diameter of 8 mm were put above the embryo's blood arteries. 20 µl of PBS and Primary OC-derived exosomes were added to each O-ring as a control and test respectively. The eggs were incubated for 24–48 h after being covered in sterilised parafilm. The O-ring was removed after the incubation time and blood vessels were imaged. Wimasis Software (Onimagin Technologies SCA, Spain) was used to analyse the vessel density in control and test samples for both 24 h and 48 h of incubation.

### Statistical analysis

GraphPad Prism was used to conduct the statistical analysis. For the comparison of two sample groups, Student's t-tests were used. When *P* ≤ 0.05, differences were considered statistically significant.

## Results

### Identification of DEGs and Hub genes related to lymph node metastasis

GEO databases (https://www.ncbi.nlm.nih.gov/gds) were used to gather microarray datasets that included Primary OC with and without LN metastases. With Gene Spring software, the data from several series were analyzed as part of a single experiment. To analyse shared up-and down-regulated genes across all of these datasets, a Venn diagram was created.

**Figure 2:**
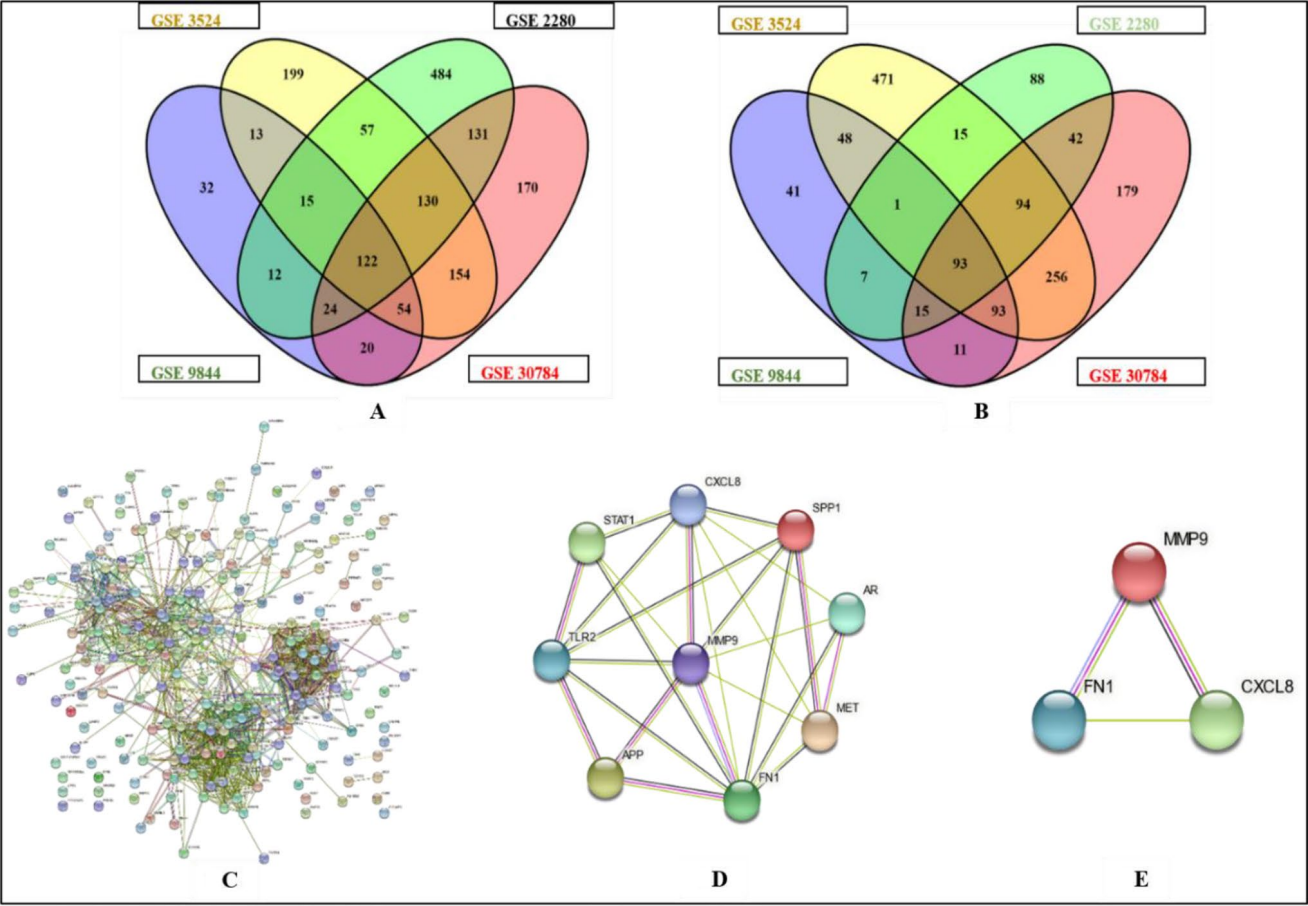
DEGs and Hub genes Identification: (**A**) and (**B**) Venn diagram representing the common up and down regulated genes respectively among the four datasets. The numerals represent the number of genes that were differently expressed in the datasets represented by the overlapped circles. (**C**) STRING output showing interaction of the common 122 up and 92 down regulated genes. (**D**) Identification of Hub genes using CytoHubba. (**E**) Top 3 hub nodes as hub genes found on the basis of Bottleneck, Degree and closeness.

All four datasets of primary and LN metastasis shared 122 genes that were upregulated and 92 genes that were downregulated (Fig. 2A,B respectively). Additionally, the PPI network comprising merged up-and down-regulated genes was constructed using the STRING tool for hub gene identification (Fig. 2C), and hub nodes were identified using CytoHubba (Fig. 2D). We selected the top 3 hub nodes as hub genes (Fig. 2E) for future investigation based on bottleneck, degree, and closeness. According to the study's findings, the Hub gene for primary OC to LN metastasis includes CXCL8, MMP9, and FN1.

### Gene ontology (GO) analysis and signaling pathway enrichment of DEGs

**Figure 3:**
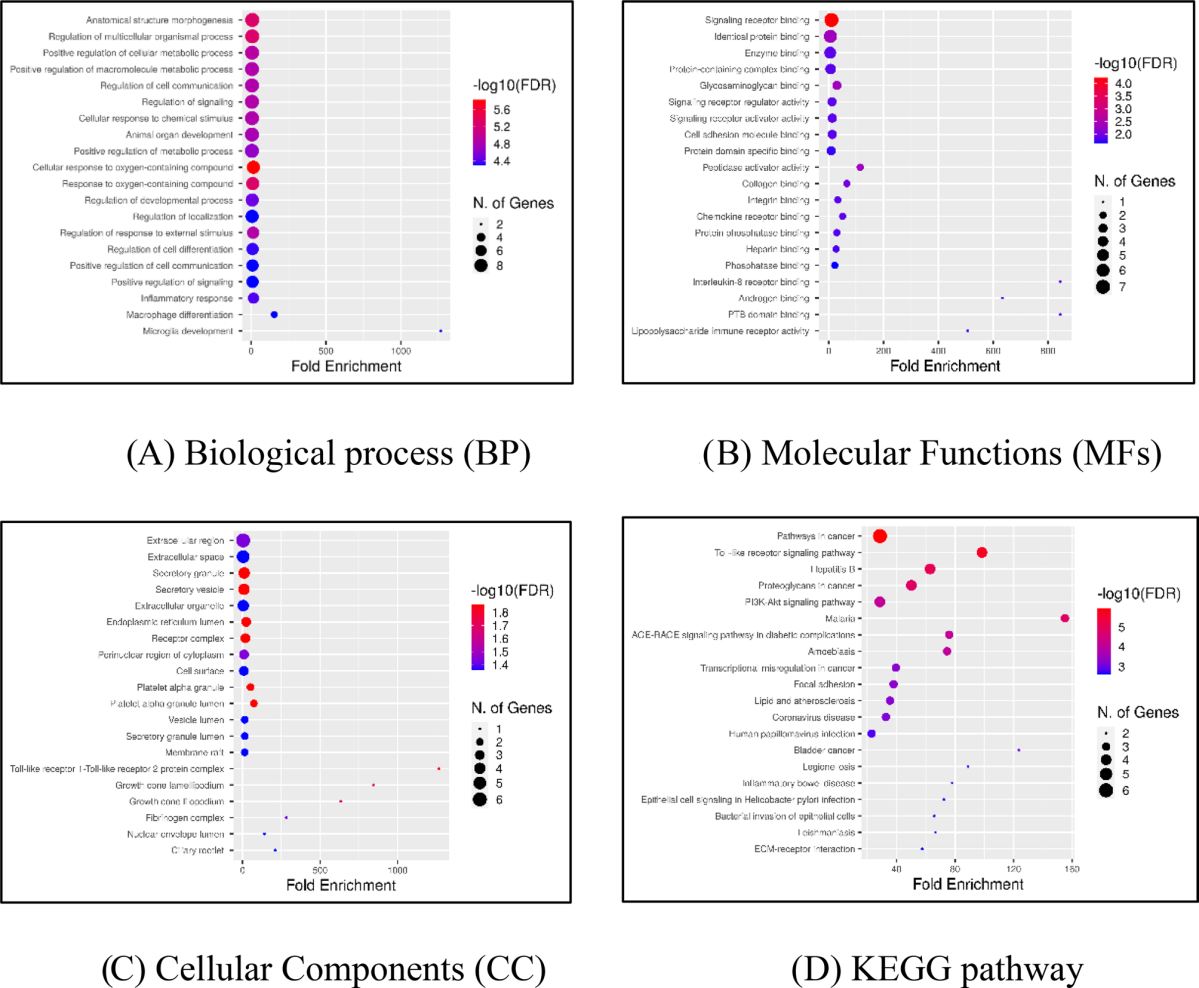
GO and KEGG pathway analysis using ShinyGO tool. (**A**), (**B**) and (**C**) represents the significant role of hub genes in Biological process (BP), Molecular Functions (MFs) and in Cellular Components (CC). (**D**) represents the KEGG pathways associated with DEGs are mainly Cancer related pathways.

For assessing gene data sets, gene ontology offers a descriptive background as well as functional annotation and classification. Most BPs associated with the control of multicellular organismal activities, cellular metabolic processes, cell communication, and signalling were enriched in DEGs (Fig. 3A). MFs showed that DEGs were primarily linked to enzymes, identical proteins, and signalling receptor binding (Fig. 3B). According to the CC of GO, DEGs were primarily located in extracellular space and secretory vesicles (Fig. 3C).

**Figure 4:**
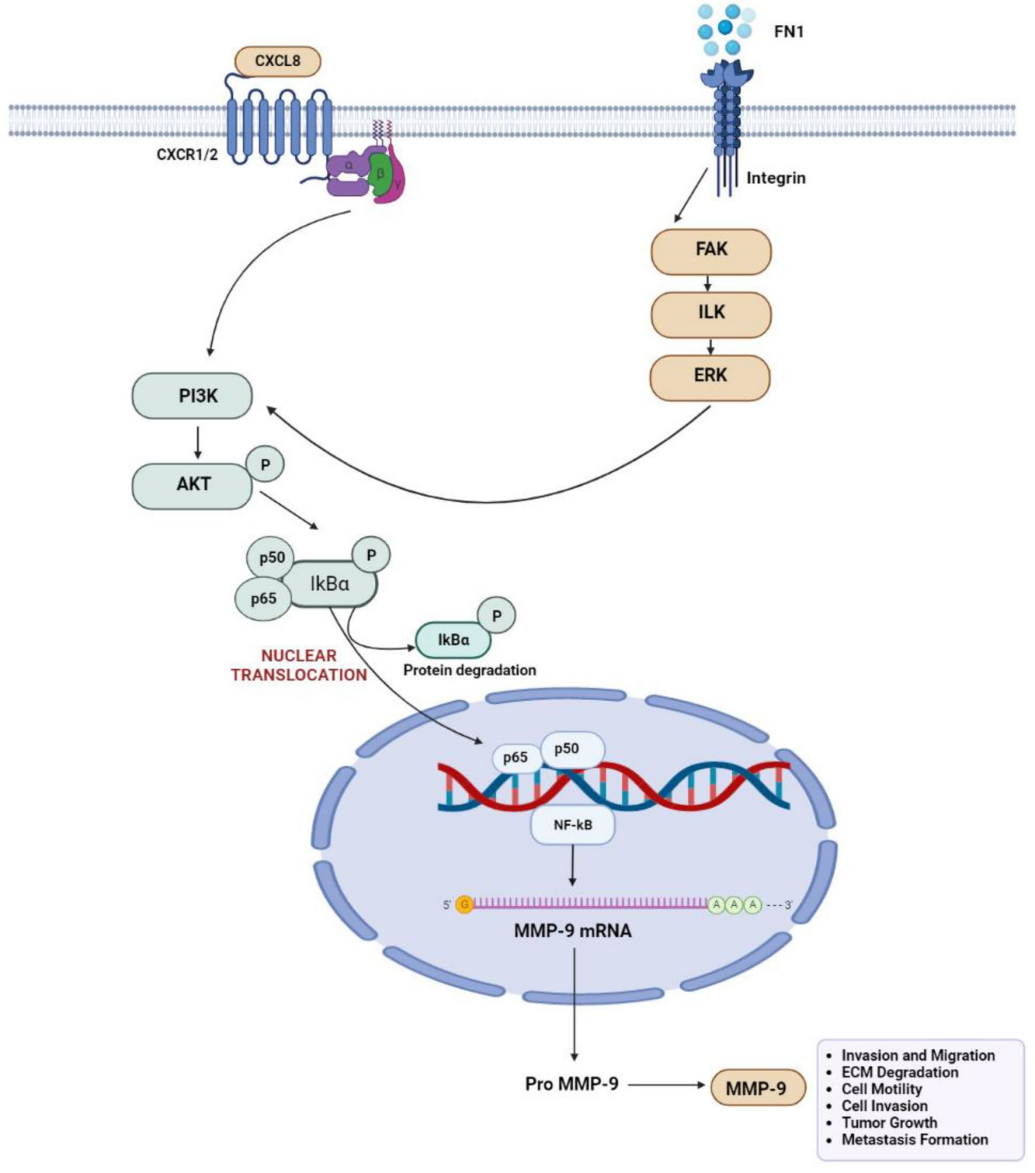
Molecular mechanisms connected with metastases progression. The cross-talk between the various signal transduction pathways shown in the figure is represented by the main active genes MMP9, CXCL8, and FN1, which control processes like ECM degradation, cell motility, cell invasion, tumour growth, and metastasis.

Further we used the BioRender software (https://www.biorender.com/) to analyze the most important and prevalent gene networks in order to better understand the mechanistic basis by which the three key genes modulate multiple downstream signalling pathways and trigger lymph node metastasis. By enhancing our understanding of cancer pathways, we were able to pinpoint pathways that were developed or overrepresented in the common metastatic signature. The key active genes MMP9, CXCL8, and FN1, which regulate activities such as ECM breakdown, cell motility, cell invasion, tumor growth, and metastasis, are a representation of the cross-talk between the numerous signal transduction pathways depicted in Fig. 4. The FAK/ILK/ERK/PI3K/NFB pathways are activated when FN1 binds to integrin, which causes overexpression of MMP-2 and MMP-9 and the destruction of the extracellular matrix (ECM) as well as the invasion of cancer cells. Our information indicates that these pathways were important for the development of lymph node metastasis.

### Characterisation of serum derived exosomes

Exosomes were isolated from serum of OC patients with and without LN metastasis and were characterised for particle size distribution and exosomal marker identification. NTA, FE-SEM and Flow Cytometry were used to characterise the isolated vesicles.

**Figure 5:**
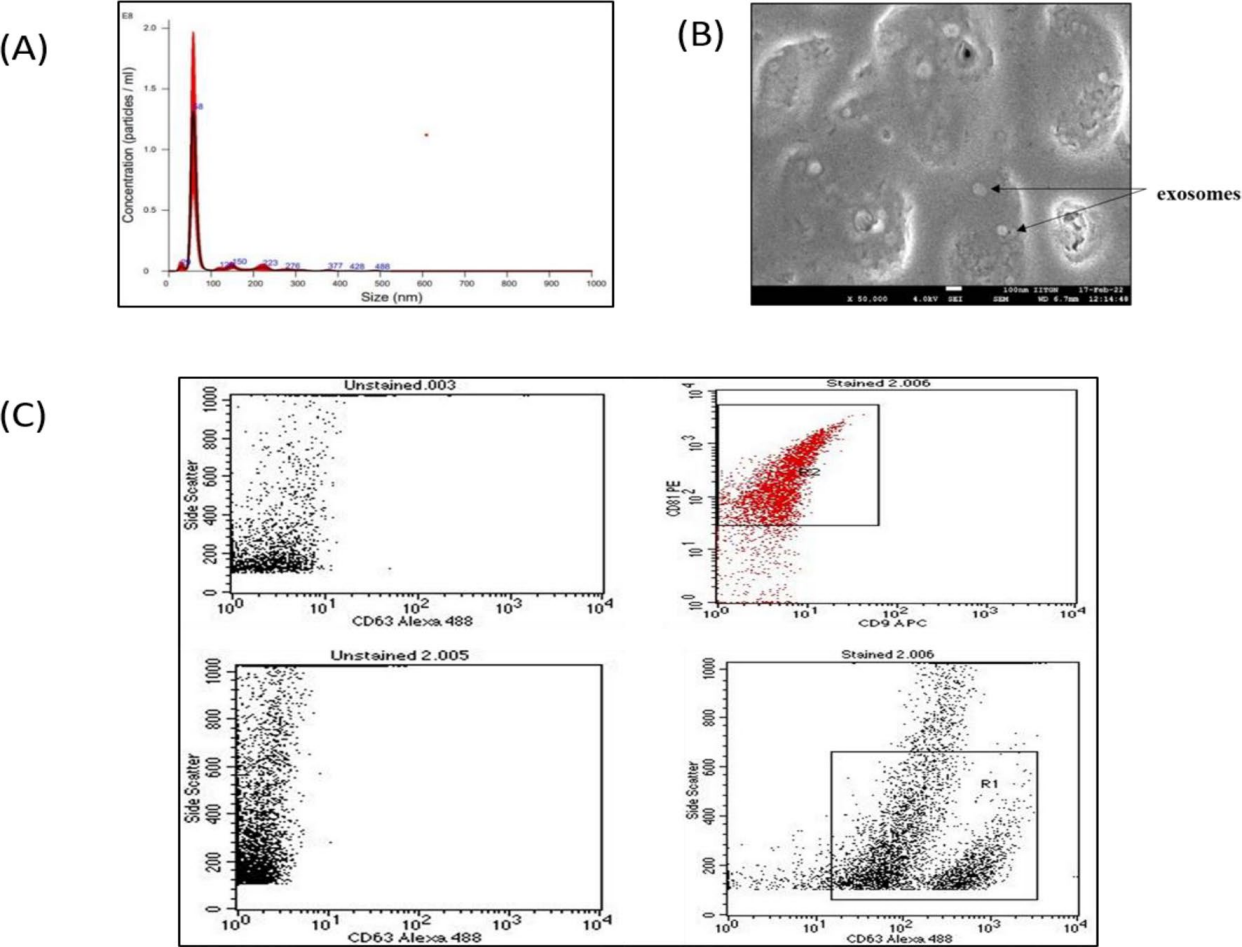
Characterisation of Exosomes (**A**) NTA analysis of isolated vesicles confirms the particle size and concentration (**B**) FE-SEM results represents the exosomes had a diameter of 30–100 nm. (**C**) Exosomal markers (CD9, CD63, and CD81) were analysed using Flow-cytometer. CD81 and CD9 were found to be abundant.

The heterogenous size of exosomes were observed in NTA with the maximum size comprised of 58 nm which confirms that the isolated entity were exosomes (Fig. 5A). Figure 5B shows the results of FE-SEM, exosomes were all spherical and had a diameter of 30–100 nm. Before the SEM study, agglomeration developed as a result of the drying process. Flow cytometry data further demonstrated that exosomes were positive for three distinct exosomal markers: CD63, CD81, and CD9 (Fig. 5C).

### Comparison of exosomal and tumor mRNA expression of MMP9, CXCL8 and FN1 between primary OC with and without LN metastasis

**Figure 6:**
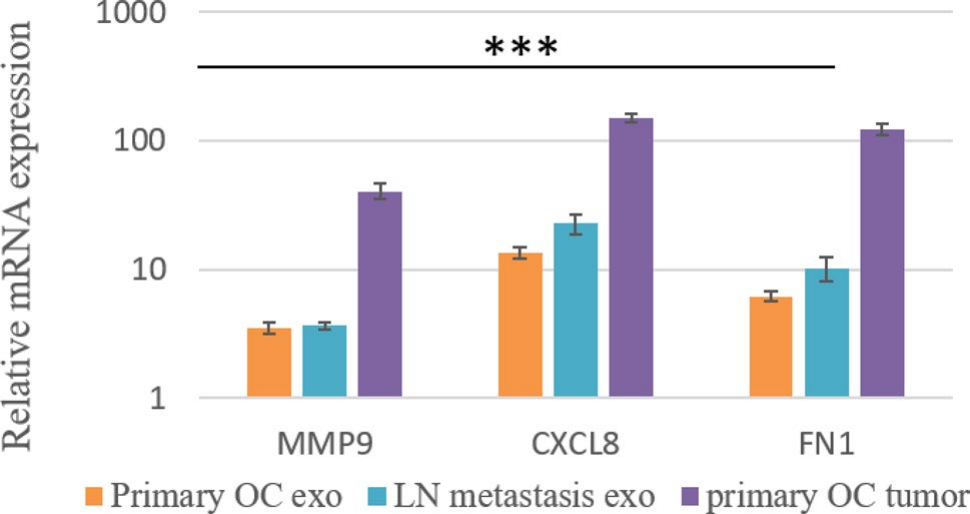
Quantitative real time PCR results of FN1, MMP9 and CXCL8. Expression of these genes were normalised against GAPDH expression. The statistical significance of differences was calculated by one-way ANOVA statistic test ***p < 0.05.

Figure 6 illustrates the pattern of gene expression that can be used to identify initial OC tumors from LN metastatic malignancies. Exosomes and tumor mRNA from primary OC and LN metastatic patients were analyzed for their quantitative gene expression patterns of MMP9, CXCL8, and FN1.

In the group of primary OC tumors, primary OC exosomes, and exosomes originating from LN metastatic patients, these genes were significantly upregulated.

Moreover, LN metastatic exosome was shown to significantly up-regulate the mRNA expression of FN1, MMP9, and CXCL8 (> twofold, *p* ≤ 0.05, one-way ANOVA statistical test) in comparison to primary OC-derived exosome.

### ROC curve analysis and Intercorrelation among the gene

To discover whether variations in gene expression are associated, we analyzed the ratios between the expression levels of numerous. In tissue samples FN1 was strongly correlated with MMP9 (*p* = 0.000) and CXCL8 (*p* = 0.003) (Table 3). Moreover, MMP9 was found to be significantly correlated with CXCL8 (*p* = 0.005).

**Table 3:** Intercorrelation among the DEGs.

			FN1	MMP9	CXCL8
Spearman’s rho	FN1	Correlation Coefficient		0.717**	0.624**
		Sig. (2-tailed)		**0.000**	**0.003**
		N		20	20
	MMP9	Correlation Coefficient			0.600**
		Sig. (2-tailed)			**0.005**
		N			20

Intercorrelation were carried out using Sperman’s correlation and it was found to be significant if *p* < 0.01. Results indicates FN1 is strongly correlated with MMP9 and CXCL8. Further, MMP9 is significantly correlated with CXCL8.

**Correlation is significant at the 0.01 level (2-tailed).

Significant values are in bold.

**Figure 7:**
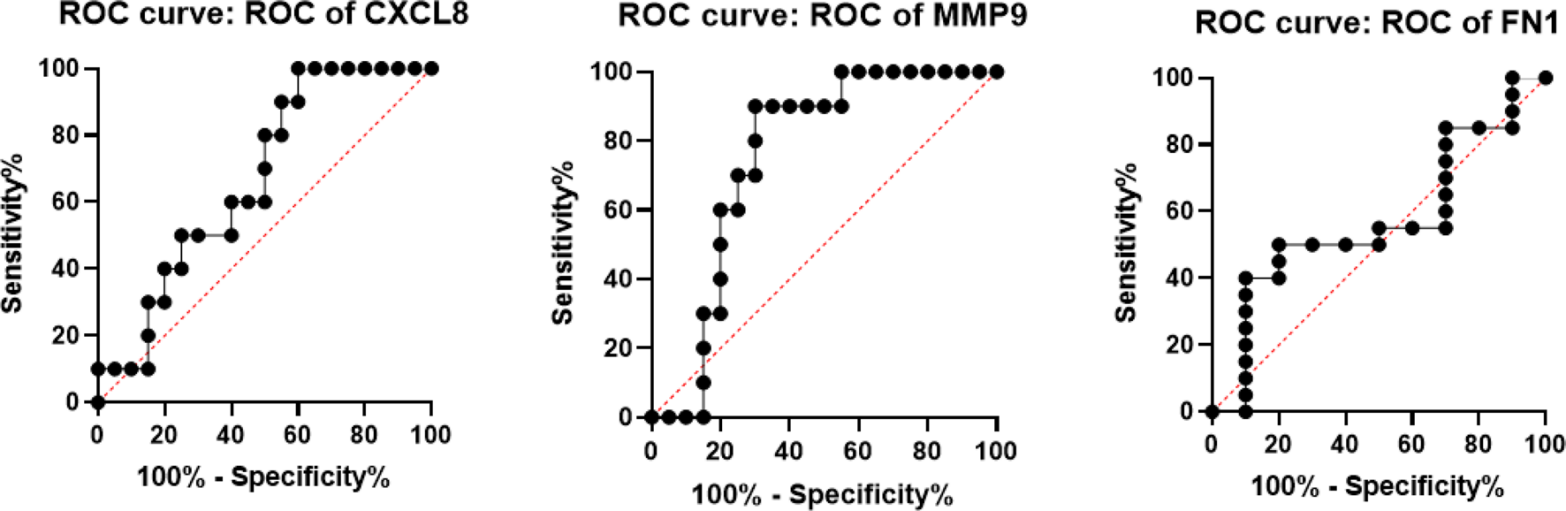
ROC curve. Receiver operating characteristic curve analysis of FN1, CXCL8 and MMP9. Area Under Curve for CXCL8, MMP9 and FN1 was 0.670, 0.755 and 0.570 respectively. Further, MMP9 was found to be significant (*p* = 0.0249).

Furthermore, ROC curve analysis was used to assess the predictive value of FN1, CXCL8, and MMP9 as metastatic biomarkers. The AUC for CXCL8, MMP9, and FN1 were 0.670, 0.755, and 0.570, respectively, and were within the range of 0.5 to 1.0. Also, MMP9 was determined to be significant (*p* = 0.0249) (Fig. 7).

### Validation of Hub genes using HPA database

**Figure 8:**
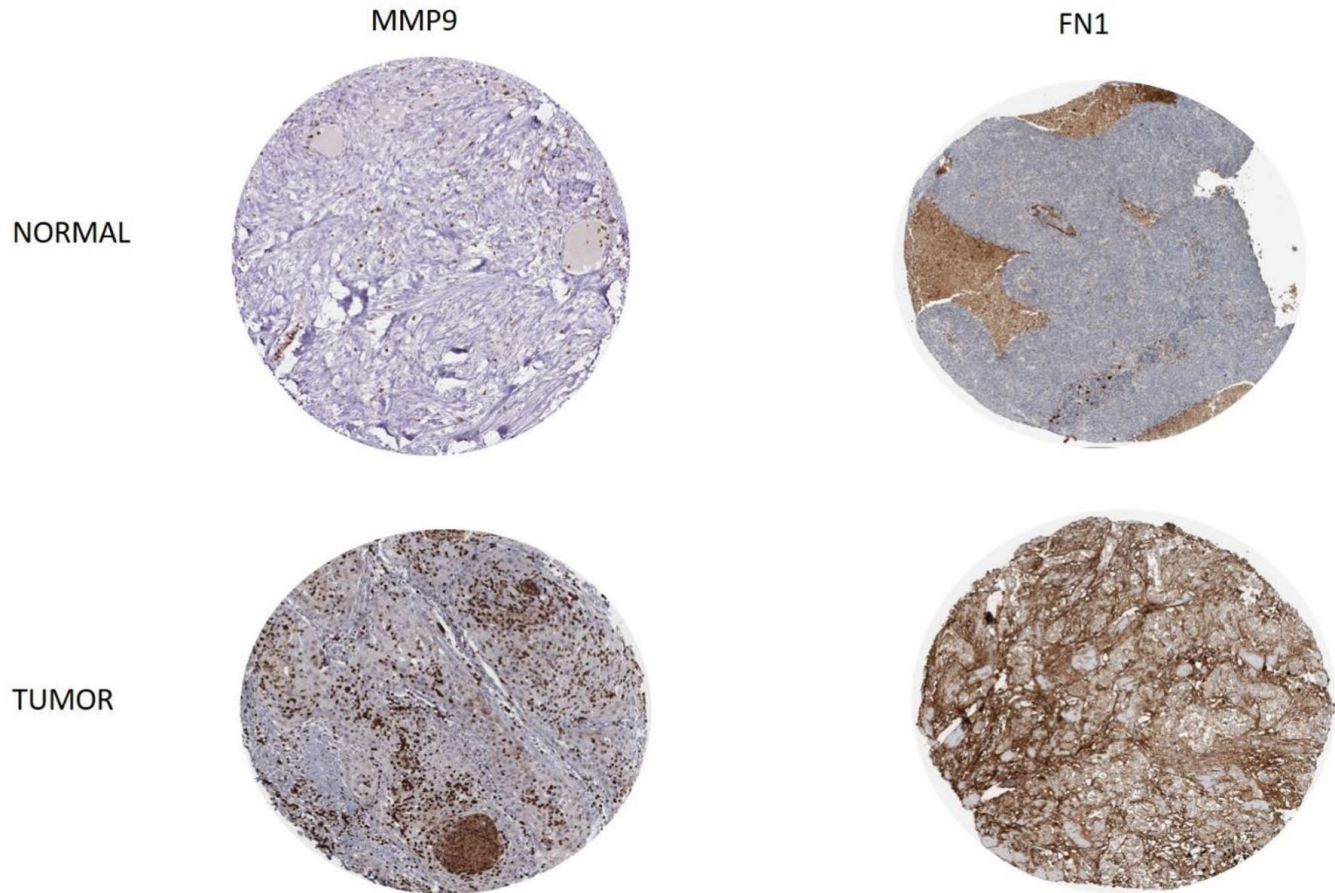
Investigation of differentially expressed target genes protein level in HPA database (magnification: 200 ×).

The protein level of the hub genes was further explored through HPA database, MMP9 and FN1 expression levels in OC tissue were overexpressed than those in normal tissue, indicating potential for oncogenesis. Protein expression level of CXCL8 was not evaluated due to unavailability of information in the Human Protein Atlas database (Fig. 8).

### CAM angiogenesis assay

**Figure 9:**
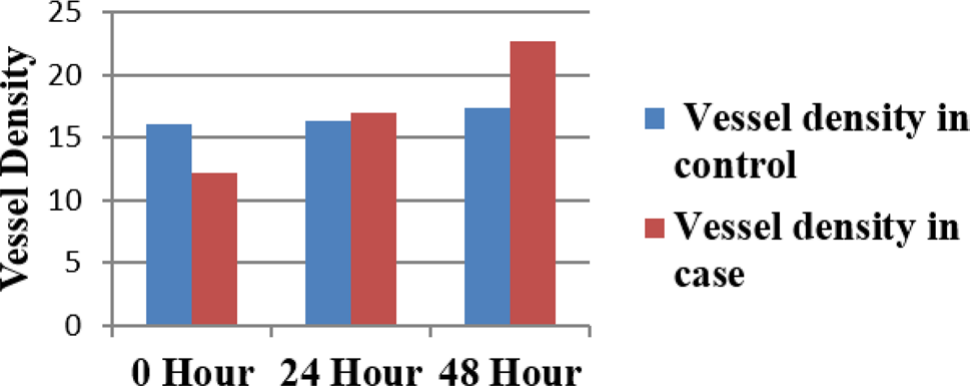
Bar graph represent the effect of primary OC derived exosomes on CAM layer. The increasing trend of vessel density was observed on CAM inoculated with primary OC derived exosomes.

**Figure 10:**
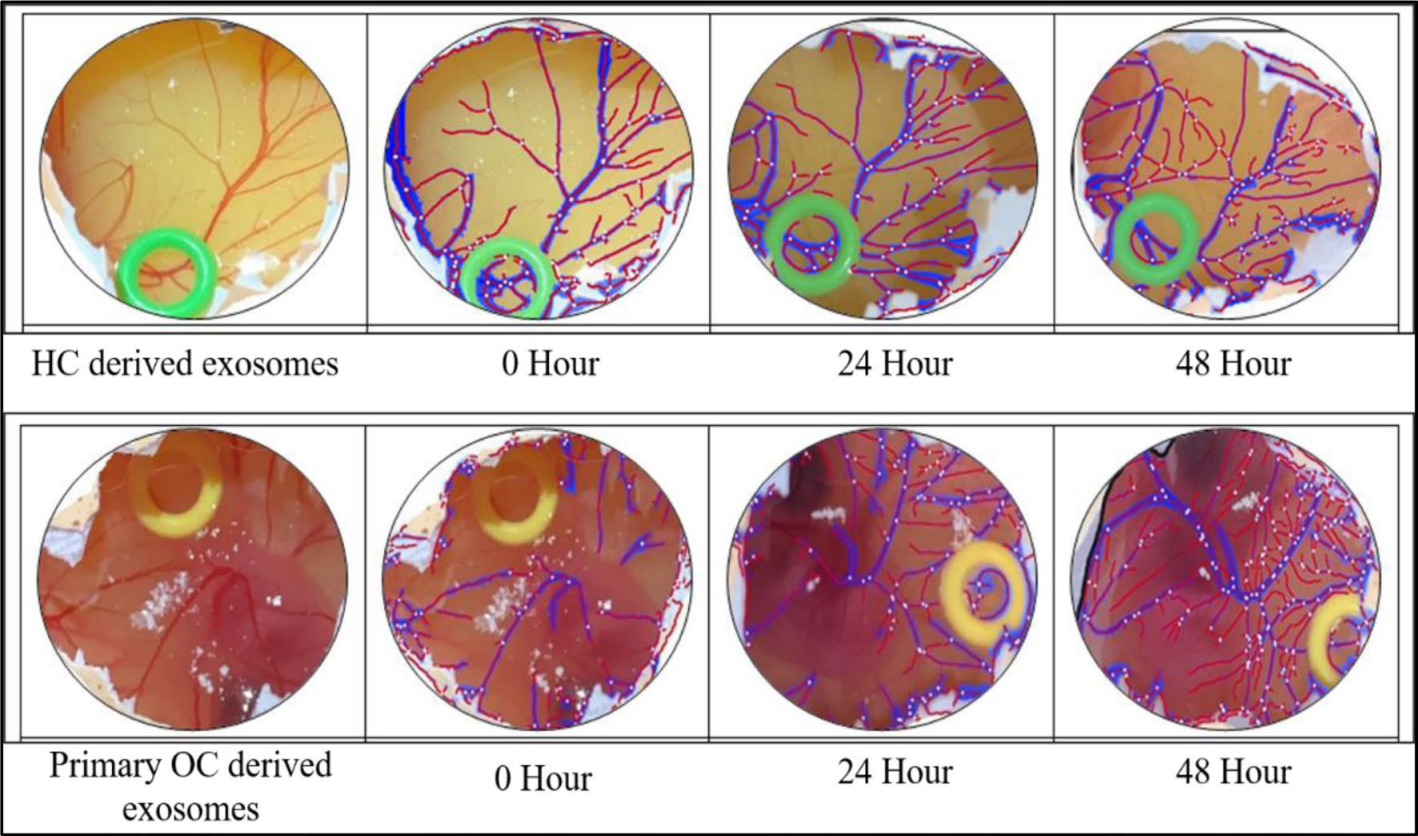
Analysis of images was done through online WIMASIS software (Onimagin Technologies SCA, Spain) for time interval of 0-h, 24 h and 48 h. Blue and Red color in the images shows the total vessel length and vessel density respectively. Samples were loaded on this silicon O ring.

The Chorioallentoic membrane (CAM) angiogenesis experiment was utilized to investigate the involvement of exosomes in metastasis. Images were acquired at 0-h, 24 h, and 48-h intervals after exosomes were loaded on the CAM layer. Wimasis Software was used to analyse the images. Exosomes from primary OC patients were inoculated into the embryos, which resulted in a significant increase in vascular density (Table 4 and Fig. 9, 10).

**Table 4:** Vessel density in control and cases.

	0 h	24 h	48 h
Vessel density in control	16.1	16.3	17.4
Vessel density in case	12.2	17	22.7

## Discussion

Oral cancer is the sixth most common type of cancer in the world^23^. It is a far bigger issue in India than in the West. Wide exposure to risk factors such as smoking or chewing tobacco, chewing betel nut or betel quid, drinking alcohol, infection with the Human Papilloma Virus, any family history of the disease, and poor oral hygiene are accountable for OC^24^. More than half of OSCC patients experience at least one recurrence, and 90% of recurrent cases happen within two years of the initial therapy^25^. Around two-thirds of OSCC patients are found to be at an advanced stage when they are first diagnosed. It should be noted that lymph node metastasis, the most common kind of OC metastasis, is a substantial and independent prognostic factor influencing the effectiveness of therapy. Those with OSCC who had lymph node metastases had a 5-year survival rate that was about half that of those who did not have the condition^26^. Because of this, reducing the fatality rate among those with oral cancer requires early detection. Therefore, early identification is critical in decreasing the death rate of individuals with oral cancer. While numerous biomarkers have been developed in recent years to predict lymph node metastasis, there is currently no marker or panel of markers that can be extensively used in clinical management. So, there is a significant demand for non-invasive, rapid, and easy oral cancer diagnostic treatments. Hence, the main goal of the current investigation was to identify clinically meaningful lymph node metastasis biomarkers using a liquid biopsy approach.

An effective way to identify potential cancer biomarkers is to molecularly characterize tumours using high throughput global transcriptome profiling. The best use of publicly accessible datasets is required for global profiling research, as is the discovery of a functionally relevant, robust subset of biomarkers that can be verified for their clinical application. In this context, meta-analysis, a systematic approach that enables data analyses from independent research and merges them using standard statistical pipelines, is of utmost value. Global variations in gene expression have been compared across samples of non-metastatic and metastatic human tumors using microarray analysis^27–29^. With the use of bioinformatics analysis of the gene expression profile of metastatic and non-metastatic lymph nodes and original tumors, we identified a novel oral cancer metastatic gene signature, FN1, CXCL8, and MMP9. Furthermore, the notable upregulation of these genes in exosomes generated from individuals with metastatic disease raises the possibility that liquid biopsies may be useful in predicting the prognosis of OC patients. Exosomes, which have a double-layer sac-like form with a diameter of 30–150 nm, have become a novel medium for liquid biopsies of tumors because of their distinctive expression patterns and relatively stable contents^30^.

The intercorrelation of these genes suggests a possible target for therapy monitoring and prognosis of OC patients. Fibronectin 1 (FN1) is comprised of various identical repeating units and is involved in cell motility, proliferation, and differentiation, as well as matrix formation and cell adhesion processes^31^. It has been studied that cell migration is influenced by FN1, which mediates cell-to-cell and cell matrix adhesion^32^. Overexpression of FN1 promotes tumour cell adhesion and aggregation through influencing tumour cell motility, differentiation, and proliferation^33^. FN1 also limits tumour cell migration and promotes tumour metastasis by facilitating intercellular and cell matrix adhesion. Morita et al.^34^ observed that FN1 overexpression promotes the progression of OSCC and lymph node metastases by increasing the production of vascular endothelial growth factor C Several earlier research have shed light on the relevance of FN1 as a new biomarker for OSCC. Chai et al.^35^ found that OSCC patients with lymph node metastasis had higher serum fibronectin levels than those without. Additionally, FN1 inhibits tumour cell migration and promotes tumour metastasis by mediating intercellular and cell matrix adhesion^36^. Using GEO2R online tool for the identification of DEGs, Xu XL, Liu H et al. have found SPP1 and FN1 that may be associated with the occurrence, lymph node metastasis and malignant progression of TSCC^32^.

Degradation of the extracellular matrix (ECM), which is a hallmark of cancer, is mainly caused by proteinases. Matrix metalloproteinases (MMPs), particularly MMP-9, are linked to this degradation in oral cancers. MMPs release various substances from their cryptic sites, including cytokines, and break down the ECM, allowing cancer to spread. These factors control angiogenesis, migration, proliferation, and invasion to alter cell behaviour and accelerate the progression of cancer. Early metastases are frequently developed in oral cancer, and increased MMP-9 expression is linked to a poorer prognosis. Thus, the role of MMP-9 was purely associated with the degradation of the ECM, which led to the enhancement of carcinoma cell invasion^37^. In the study by Atla et al.^38,39^, the MMP9 level was found higher in 70% of the cases with lymph node metastases and at low levels in 30% of the cases without lymph node metastases.

CXCL8, a multifunctional proinflammatory chemokine, is increased in both the tumour and tumor-derived microenvironment, where it regulates proliferation, migration, angiogenesis, metastasis, and chemotherapeutic resistance^40^. Further, CXCL8 has been shown to promote the synthesis and release of MMP2 and MMP9, indicating that invasiveness and extracellular matrix remodelling can be influenced by CXCL8^41^. In various xenograft and orthotopic models, high levels of CXCL8 expression in tissue may correlate with tumorigenicity, angiogenesis, and metastasis^42^. A high expression level in a tumour sample in humans may be related with a poor prognosis when stratified by tumour stage and pathology classification^43^. All this suggests that CXCL8 can be used as a cancer biomarker for prognosis and prediction to identify a more aggressive phenotype. As a result, inhibiting CXCL8 may be a potential therapeutic method for targeting the tumour and its associated milieu^44^.

To the best of our knowledge, no one has previously investigated the expression levels of the aforementioned gene panel in exosomes isolated from patients with OC to LN metastasis along with the bioinformatics approach. Our significant gene signature identification demonstrated increased serum exosome efficacy in early detection and was clinically associated with intracellular communication in the formation of the premetastatic niche. Exosomes transport genetic materials such as microRNAs (miRNAs), mRNAs, and proteins that are similar to those found in parent cells^45^. Exosomal microRNAs have previously been found to control the expression of FN1 in mesothelial cells^46^. MMPs promote OSCC metastasis by degrading ECMs^47^. Specifically, MMP9 was found to be highly upregulated in tumour cell exosomes^48^. It has previously been shown that hepatocellular carcinoma-derived exosomes could increase MMP9 secretion in hepatocytes 5^49^ Also, Zhang et al.^50^ have studied the potential of the expression levels of MMP-9 and CXCL8 in the blood for the early screening of breast cancer.

There are some limitations of our study. The sample size was relatively small. As a result, the association between exosomal FN1, MMP-9, and CXCL8 expression levels and the risk of OC LN metastasis should be investigated further with a larger sample size. Also, a validation cohort is required to validate the diagnostic value of exosomal FN1, MMP-9, and CXCL8 in early detection of LN metastasis. Due to the unavailability of data, the clinical survival of data from tumour tissues and exosomes was not evaluated. Furthermore, without a biomarker specifically for cancer exosomes, we were unable to characterise the biological function of tumor-derived exosomes.

The prediction accuracy of the discovered gene panel in this study was assessed using ROC curve analysis. Moreover, the chick CAM model provides quick, simple, and inexpensive tissue reaction screening to exosomes derived from an OC^51^. Also, the current study offered the first proof that exosomes from OC patients who had lymph node metastasis might be significant in the formation of the angiogenesis in CAM model. Our findings suggest that, while some genes were commonly modulated, the effects were significantly amplified in recipient cells by cancer exosomes. Further, the mRNA in exosomes can be helpful biomarkers of OC development and metastasis and it is very effective in detecting and predicting lymph node metastases using liquid biopsy based approach.

## Conclusion

The meta-analysis of publicly available datasets using appropriate statistical tools provides a cost-effective method of generating novel hypotheses that may then be verified using more sensitive molecular approaches. The current study revealed FN1, MMP9, and CXCL8 as potential biomarkers for identifying and predicting lymph node metastases. Additional evidence for the possible use of liquid biopsies in the diagnosis and prevention of early metastases in OSCC comes from the significantly elevated expression of these genes in exosomes. The practical application of these biomarkers will, however, require more clinical investigation.

### Supplementary Information

Supplementary Information.

## Data Availability

The datasets generated during and/or analysed during the current study are available from the corresponding author on reasonable request. GSE9844—https://www.ncbi.nlm.nih.gov/geo/query/acc.cgi?acc=GSE31056. GSE30784—https://www.ncbi.nlm.nih.gov/geo/query/acc.cgi?acc=GSE30784. GSE3524—https://www.ncbi.nlm.nih.gov/geo/query/acc.cgi?acc=GSE3524. GSE2280—https://www.ncbi.nlm.nih.gov/geo/query/acc.cgi?acc=GSE2280.
